# A single blind randomized controlled trial of cognitive behavioural therapy in a help-seeking population with an At Risk Mental State for psychosis: the Dutch Early Detection and Intervention Evaluation (EDIE-NL) trial

**DOI:** 10.1186/1745-6215-11-30

**Published:** 2010-03-22

**Authors:** Judith Rietdijk, Sara Dragt, Rianne Klaassen, Helga Ising, Dorien Nieman, Lex Wunderink, Philippe Delespaul, Pim Cuijpers, Don Linszen, Mark van der Gaag

**Affiliations:** 1 VU University and EMGO+ Institute of Health and Care Research Amsterdam, Department of Clinical Psychology, Van der Boechorstraat 1, 1081 BT, Amsterdam, the Netherlands; 2 Academic Medical Centre (AMC), Adolescent psychosis clinic, Department of Psychiatry, Meibergdreef 5, 1105 AZ, Amsterdam, the Netherlands; 3 GGz Rivierduinen, Department of Children and Adolescent care, Albinusdreef 7, 2333 ZA, Leiden, the Netherlands; 4 Friesland Mental Health Service, Department of Education and Research, Jacob Catsstraat 2, 8913 CM, Leeuwarden, the Netherlands; 5 Maastricht University, Department of Mental Health Services Research and Development, Vijverdalseweg 1, 6226 NB, Maastricht, the Netherlands; 6 Mondriaan Institute, Department of Integral health care Maastricht/Parkstad, Vijverdalseweg 1, 6226 NB Maastricht, the Netherlands; 7 Parnassia Psychiatric Institute, Department of Psychiatry, Prinsegracht 63/65, 2512 EX, The Hague, the Netherlands

## Abstract

**Background:**

Psychotic disorders are a serious mental health problem. Intervention before the onset of psychosis might result in delaying the onset, reducing the impact or even preventing the first episode of psychosis. This study explores the effectiveness of cognitive behavioural therapy (CBT) in targeting cognitive biases that are involved in the formation of delusions in persons with an ultra-high risk for developing psychosis. A single blind randomised controlled trial compares CBT with treatment as usual in preventing or delaying the onset of psychosis.

**Method/design:**

All help seeking patients aged 14 to 35 years referred to the mental health services in three regions in the Netherlands are pre-screened with the Prodromal Questionnaire during a period of two years. Patients with a score of 18 or more on the sub-clinical positive symptoms items (45 items in total) will be assessed with the Comprehensive Assessment of At Risk Mental State (CAARMS). In a different pathway to care model all referrals from the mental health services in Amsterdam to the specialized psychosis clinic of the Academic Medical Centre in Amsterdam are also assessed with the CAARMS. The primary outcome is the transition rate to psychosis according to the CAARMS-criteria. Group differences will be analysed with chi-square tests and survival analyses.

**Discussion:**

CBT is a highly tolerated treatment. The psycho-educational CBT approach may prove to be a successful strategy since most people with an At Risk Mental State (ARMS) are distressed by odd disturbing experiences. Giving explanations for and normalising these experiences may reduce the arousal (distress) and therefore may prevent people from developing a catastrophic delusional explanation for their odd experiences and thus prevent them from developing psychosis.

Screening the entire help-seeking population referred to community mental health services with a two-stage strategy, as compared with traditional referral to a specialist clinical psychosis centre, might detect more ultra-high-risk (UHR) patients. This type of screening could be implemented in mental health care as routine screening. The trial is registered at Current Controlled trials as trial number ISRCTN21353122.

## Background

In recent decades, the number of studies on early detection and intervention in psychosis has increased exponentially. In the UK [1,2], Australia [3], Norway [4] and the Netherlands [5], specialized programs have been developed for first psychotic episode patients. An unexpected finding in a seminal study was the identification of a group of help-seeking young people with sub threshold psychotic symptoms that made a transition to psychosis [6]. This phase prior to a first episode of psychosis, retrospectively called the prodromal period, begins with the first changes in behaviour and lasts until the onset of the first psychotic episode [7]. The prodromal period can be characterized by various mental state features, including non-specific symptoms such as depressed mood and anxiety, negative signs and symptoms as well as sub-threshold or attenuated psychotic symptoms [6]. However, the term "prodromal" is not always correct in prospective investigations. After all, only in the at risk people who actually develop a full-blown psychosis, the symptoms can be defined as 'prodromal'. The majority of individuals who share the same sub-clinical symptoms will recover spontaneously or have persistent sub-clinical symptoms [8] without a transition into psychosis. Instead of putatively prodromal, in prospective studies the subjects are referred to as being at ultra high risk (UHR) or having an At Risk Mental State (ARMS) of developing psychosis.

Yung and McGorry were the first to develop operational criteria to detect people at ultra high risk of developing psychosis, resulting in the Comprehensive Assessment of At Risk Mental State (CAARMS) scale [8]. At first about 40% of the people with ARMS made a transition to psychosis within a year [8,9]. However, more recent studies find lower transition rates (declining to only 16% transition to psychosis at 24 months) [10,11].

The identification of people at high risk for developing psychosis has been replicated in several studies in Australia, Europe and the USA [12-15]. People with an ARMS experience (mild) symptoms, are often help-seeking and frequently suffer psychosocial impairment and disability [6]. Therefore one important aspect of the ARMS is that it is a status in which an intervention could be delivered to prevent transition. Detection of people with ARMS is therefore required. When people do make a transition then the potential duration of untreated psychosis is probably short. Previous research has shown that a shorter delay in treating the first episode of psychosis is associated with a better outcome [16]. Intervening before the first psychotic episode can thus be a valuable treatment option. The aim is reduction of high risk symptoms and to try to prevent or delay the onset of psychosis as well as a reduction of suffering from psychological impairments [13].

Several intervention studies have been performed in the ARMS-group [3,12,14,15,17]. Interventions in these studies include prescribing drugs (anti-psychotics or others), cognitive behavioural therapy (CBT) or a combination of these treatments. One study delivers a complete treatment package to the patients [15]. These studies suggest that an intervention may reduce the transition to psychosis in the short term. Recently published reviews on interventions in people at high risk for developing psychosis concluded that the effects of interventions are currently indecisive, implying that more research is necessary [18,19].

### Aim

The objective of this study is to test a manualised CBT aimed at reducing the transition rate to psychosis and to reduce the number of patients with persistent ARMS-symptoms. This CBT is largely based on the work of French and Morrison [14,20,21] who developed a promising intervention for reducing or postponing the transition to psychosis in the short term. The intervention is enriched with information on cognitive biases and exercises to learn to correct these biases.

## Methods/Design

### Design

This study is a randomized controlled trial comparing treatment as usual (TAU) with an add-on cognitive behavioural therapy (CBT) targeted at the prevention of psychosis. The main outcome measure is the number of participants who develop florid psychosis within eighteen months. The secondary outcome is the reduction of the persistence of sub clinical symptoms.

The assessors are blinded to the research condition by not being able to check the patient status. The success of blinding is checked by having the assessors guess the presumed condition of the subject at each major assessment.

The design of this study has been approved by the Dutch Union of Medical-Ethics Trial Committees for mental health organizations. The trial will be conducted in compliance with the 'Declaration of Helsinki' (amendment of Edinburgh, 2000).

### Participants

All patients aged 14 to 35 years referred to the mental health services in The Hague, Rivierduinen (Leiden and surroundings) and the province of Friesland in the Netherlands will be pre-screened with the Prodromal Questionnaire [22] over a period of two years. Patients with a score above the cut-off point of 18 on the positive symptom subscale (45 items in total) will be assessed with the CAARMS [8]. Furthermore, all patients with a suspicion of a psychotic development referred by the mental health services in Amsterdam to the specialized early psychosis clinic of the department of Psychiatry at the Academic Medical Centre will be assessed with the CAARMS. Participants are eligible if the following criteria are met: a) age 14 to 35 years; b) a genetic risk or CAARMS-scores in the range of At Risk Mental State (See table 1, 2, 3); and c) an impairment in social functioning, (a SOFAS- score [23] of 50 or less and/or a drop in SOFAS score of 30%.). Patients are excluded if they meet any of the following criteria: a) current or previous usage of antipsychotic medication more than 15 mg Haloperidol equivalent; b) severe learning impairment; c) problems due to organic condition; d) insufficient competence in the Dutch language; e) history of psychosis.

**Table 1:** group 2a Attenuated psychotic symptoms, Sub-threshold intensity

	Intensity	Frequency
Unusual Thought Content	3-5	3-6

Non-bizarre Ideas	3-5	3-6

Perceptual Abnormalities	3-4	3-6

Disorganised Speech	4-5	3-6

**Table 2:** group 2b Attenuated psychotic symptoms, sub-threshold frequency

	Intensity	Frequency
Unusual Thought Content	6	3

Non-bizarre Ideas	6	3

Perceptual Abnormalities	5-6	3

Disorganised Speech	6	3

**Table 3:** group 3, BLIPS Group

	Intensity	Frequency
Unusual Thought Content	6	4-6

Non-bizarre Ideas	6	4-6

Perceptual Abnormalities	5-6	4-6

Disorganised Speech	6	4-6

Symptoms occur less than one week and resolve spontaneously

Participation is voluntary. Informed consent is given in writing and with personal signatures. Persons under 16 years also require informed consent from a parent. Participants may withdraw their informed consent at any time, without any consequences for their treatment.

### Randomisation

Randomisation will be stratified by site, to rule out factors concerning the institutions, therapists and habitat. The random allocation lists are generated by a web-based automated randomization system. To guarantee a numeric balance across conditions the randomisation will be performed separately for each research site, in random permuted blocks of ten. The allocation list will be kept in a remote secure location and an independent person randomly allocates the included patients. Patients are randomised after providing informed consent. The randomisation status is confirmed by fax to the randomisation bureau by the different sites.

### Power calculation

We calculated power on an expected transition rate of 35 percent over eighteen months with a 50 percent reduction of transitions in the CBT-group. The sample we need for a 2-tailed test of the proportions with an alpha of .05 and a power of .80 is 2 × 93 for the reduction of the transition to psychosis and 2 × 82 for the persistence of ARMS and 2 × 91 for the transition into psychosis. A conservative estimate of the drop-out rate is twenty percent per year in schizophrenia research [24]. With an estimated 30 percent drop-out over 18 months, we decided to include 240 persons in the trial. Interventions to minimize drop-outs are flexibility to location of therapy (the appointment can be at their home-address or some times by telephone or webcam), sending Christmas- and Birthday cards every year. For the participants that end up in the CBT-treatment group there is also the possibility for web-cam therapy. All the participants that complete the study will have a financial compensation for expenses made.

### Intervention

Participants in the control condition will receive treatment as usual (TAU) for the mental problems that they are seeking help for (e.g., depression, ADHD or anxiety disorder). The subjects in the intervention group will receive TAU plus a manualised cognitive behavioural therapy (CBT). The intervention protocol, based on the protocol from the British intervention trial [20,21], is a cognitive behavioural intervention that aims to reduce symptoms, normalises psychosis-like experiences and prevents a catastrophic appraisal of the psychotic-like symptoms from occurring. The idea is that the final common pathway from ARMS to psychosis is largely based on catastrophising the psychotic-like symptoms which are then worsened by a high level of emotional arousal. When the appraisals become fixed and frightening, delusions are formed. The therapy manual in this study is enriched with eight sessions of psycho-education and behavioural experiments with cognitive biases that play a role in the development of delusions and hallucinations. Normalising the odd experiences as a result of perceptual and reasoning biases is supposed to reduce the emotional arousal and over-involvement with the experiences. Catastrophic delusional interpretation of the unsettling experiences is then probably prevented. Behavioural experiments and homework exercises teach people to tolerate psychosis-like experiences and reduce emotional discomfort. The intervention consists of a maximum of 25 sessions within a six-month period. All therapists are psychologists or consultant psychiatrists experienced in CBT with psychotic patients. They are trained in using the protocol and are offered supervision every two weeks during the course of the trial. During the two-hour supervised sessions, audio-taped sessions are discussed and rated on the Cognitive Therapist Scale [25]. Another part of the supervised sessions is dedicated to case formulation and trouble shooting of difficult cases.

### Measurements

(See also table 4 for measurement moments and instruments)

**Table 4:** measurements

Measurements	T0: baseline	T6: end of the intervention	T12: Follow up	T18: follow up	Transition
CAARMS	X	X	X	X	x

BDI	X	X	X	X	-

SIAS	X	X	X	X	-

EQ5D	x	X	X	X	-

PBIQ-R	x	X	X	X	-

MANSA	x	X	X	X	-

CDS	x			X	x

Verbal Fluency	x	X	X	X	-

Medication check	x	X	X	X	x

Blood sample	x	-	-	-	-

SCAN interview	x	-	-	-	x

PANSS	-	-	-	-	x

PSYRATS	-	-	-	-	x

Treatment check	-	-	-	-	x

CAARMS = Comprehensive Assessment of At Risk Mental State; BDI = Beck Depression Inventory; SIAS = Social Interaction Anxiety Scale; EQ5D = Euroqol-5D; PBIQ-R = Personal Beliefs about Illness Questionnaire-Revised; MANSA = Manchester Short Assessment of Quality of Life; CDS = The Calgary Depression Scale; SCAN = Schedules for Clinical Assessment in Neuropsychiatry; PANSS = Positive and Negative Syndrome Scale; PSYRATS = Psychotic Symptom Rating Scales.

Subjects participate in the study for 18 months. At baseline and at six-month intervals, participants are assessed with the full CAARMS [8]. During these major measurement sessions, participants are also assessed with secondary outcome measures and mediators and moderators. In between the major assessments participants are monitored with a subset of the CAARMS (the first four scales) to assess a possible transition to psychosis. The minor assessments are scheduled at months 2, 4, 9 and 15.

### Instruments

The following instruments are used:

1. The Prodromal Questionnaire (authorized Dutch translation by M. van der Gaag, R. Klaassen, L. Wunderink) [22] will be used to screen patients for psychosis-proneness in the general help-seeking population and is a 92-item self-reporting lifetime questionnaire, rated on a two point scale ('agree' and 'disagree'.) Of these items, 45 apply in the case of possible sub-clinical positive symptoms. When maximizing the true positive cases, Loewy et al. found a cut-off score of eight symptoms at the positive subscale predictive for an ARMS on the SIPS (a CAARMS look-alike) with a sensitivity of 90% and a specificity of 48% in a population that was referred because of suspected prodromal state [22]. Since we expect more false positives in the general help-seeking population, the cut-off score for the EDIE-screening is higher [26]. Patients are invited for a further structured clinical interview when the total score of positive symptoms exceeds 18.

2. The Comprehensive Assessment of At Risk Mental State (CAARMS [8], authorised Dutch version by M. van der Gaag, J. van der Werf, L. Wunderink, A. Malda, R. Klaassen) [8] is a semi-structured interview that assesses sub-clinical psychotic symptoms in the last year before assessment. Both intensity and frequency of the symptoms are assessed, in order to dimensionally distinguish between not at risk, ARMS and psychosis (see table 1, 2, 3). The CAARMS has a good reliability. High scores on the CAARMS are predictive for transition into psychosis with a relative risk of 12.44 (95% CI = 1.5 - 103.41, *p *= 0.0025). An ARMS predicts psychosis onset within one year with good sensitivity (86%), specificity (91%), positive predictive value (80%) and negative predictive value (94%) [9,25].

3. Drug and alcohol use are assessed with the Composite International Diagnostic Interview (CIDI) [27]. The CIDI is a comprehensive, fully standardized instrument for assessing mental disorders according to the definitions and criteria of ICD-10 and DSM-IV. Good reliability and validity of the CIDI have been reported with all Kappa coefficients above .5 for reliability and above .7 for validity [28].

4. Semantic verbal fluency is assessed with a subtest of the Groninger Intelligence Test, a test that is part of a Dutch set of intelligence tests comparable to the Wechsler [29]. Participants have to name as many animals as possible in one minute. Schizophrenia patients do have more difficulties with tests like this compared to depressed patients and healthy controls, due to cognitive problems [30] F(2,63) = 3.8 *p *< .05. A poor result on this test could be a predictor for schizophrenia [31].

5. Depression is assessed with the Dutch translation of the Beck Depression Inventory second edition (BDI-II-NL) [32]. The BDI is a 21-item self-report questionnaire, which assesses the presence and severity of depressive symptoms. The score ranges from 0 - 63; a high score reflects more severe depression. The BDI-II is positively correlated to the Hamilton Depression Scale (Pearson r = .71). Also the test-retest reliability and the internal consistency show high rates (Pearson r = .93 and α = .91 respectively)

6. The Calgary Depression Scale (CDS) [33] is an 8-item interview that assesses depressive symptoms independent of the negative symptoms of schizophrenia with a goodness-of-fit index of 0.89 and a root square residual of 0.07. The internal reliability was good (α 0.85). The CDS shows weak statistically significant associations with the negative symptoms on the PANSS (0.33)

7. The Social Interaction Anxiety Scale (SIAS) [34] is a 20-item self-report questionnaire for social anxiety. Total scores range from 0 to 80. A high score on the SIAS reflects more severe social phobia. The SIAS discriminates significantly (*p *< .001) between social anxiety and other anxieties and healthy controls. High internal reliability (α ranges from .88 - .94) and test-retest reliability (α = .92) is reported for all scales.

8. Ethnic identity is assessed with the Dutch version of the ICSEY (International Comparative Study of Ethno Cultural Youth) Scale of Ethnic and National Identity [35,36]. This is a 10-item self-report questionnaire, which assesses ethnic and national affirmation, sense of belonging and feelings about being a group member. Each item is rated on a 5-point scale, ranging from 'strongly disagree' (1) to 'strongly agree' (5). No information about reliability and validity is reported.

9. The Personal Beliefs about Illness Questionnaire-Revised (PBIQ-R) [37] assesses the subjective appraisal of the illness. It is a self-report questionnaire with five subscales: 1) loss, 2) humiliation, 3) shame, 4) attribution of behaviour to self or to illness and 5) entrapment in psychosis.

10. The Euroqol-5D [38] assesses quality of life. It is a self-report questionnaire and measures general health-related quality of life. The list contains five dimensions (mobility, self-care, usual activities, pain and anxiety/depression). Each item score ranges from no to extreme problem level. Good reliability and validity are reported for use within a schizophrenic population [38].

11. Manchester Short Assessment of Quality of Life (MANSA) [39]. The MANSA was developed as a slightly modified instrument for assessing quality of life and satisfaction with specific life domains. The self-report questionnaire contains 16 items, which are rated on a 6-point scale. High face and construct validity was reported for assessing quality of life (coefficients above .82 for all domains). The measured quality of life isn't specifically illness or symptom related and therefore could be used for persons with several mental illnesses [39].

12. Genetic material will be derived from blood or saliva.

13. The Positive And Negative Syndrome Scale (PANSS) [40] is a 30 item structured interview that was developed for the assessment of positive (7 items) and negative (7 items) symptoms as well as general psychopathology (16 items) over the past two weeks. The PANSS uses 7-point Likert type scales. A study with 101 Schizophrenia patients [33] found the three scales to be normally distributed and found evidence of reliability and stability for the positive and negative scales (α .73 and .83, *p *< .001). The general psychopathology scale has a high internal consistency (α .79, *p *< .001)

14. The Psychosis Rating Scale (PSYRATS) [41]consists of two subscales that assess auditory hallucinations (11 items) and delusions (6 items). Inter-rater reliability is good, with coefficients in the range of .79 to 1.00. Validity was checked by comparing the PSYRATS with the Psychiatric Assessment (KGV) scale and the PANSS. Significant relationships were found for hallucinations and delusional disruption reported at the KGV, PANSS and PSYRATS [41].

15. The Dutch version of the Schedules for Clinical Assessment in Neuropsychiatry (SCAN 2.1) [42] will be used to assess the DSM-IV disorder status at baseline and when a transition to psychosis occurs. The SCAN 2.1 is a semi-structured, diagnostic interview for DSM-IV and ICD-10 designed by the World Health organization and translated into Dutch by Giel and Nienhuis [42]. This interview assesses all kind of symptoms belonging to the most common Axis I disorders, like mood disorders, anxiety disorders, eating disorders, psychotic disorders and cognitive decline. The reliability of this instrument is qualified as moderate to substantial. Diagnosis and non-diagnosis were recognised with a sensitivity of 86% percent and a specificity of 99%. Test-retest reliability was significant for diagnosis (k = .64).

16. Social Demographic Questionnaire (SDQ):

The sociodemographic questionnaire is developed by the researchers to assess socio-demographic factors in our study that may play a role in the development of a psychosis based on previous research and know risk factors for schizophrenia. The items are grouped by type:

**1. General: **e.g. Current residence; Birth; Relationship status; Household; Previous residences.

**2. Education: **e.g. number of years full-time; training completed; duplication number; highest level achieved; highest level completed; total numbers of education years; special education; cito-score.

**3. Current situation: **e.g. currently education; Number of months been successful in training last year; Paid job; Number of months been working successfully last year.

**4. DSM-IV: **e.g. have you ever received a psychiatric diagnosis? If yes, what diagnosis?

**5. Medication: **e.g. have you ever received medication? If yes, what medications?

**6. Bullying: **e.g. Have you been bullied in the past; at what age it started and stopped; Seriousness of harassment?

**7. Family data (Hetero-history): **e.g. General; Educational history of family.

**8. Family history: **e.g. familial psychiatric disorders; what degree of family.

**9. Pregnancy: **e.g. drugs, alcohol, smoking, anaemia during pregnancy; unwanted pregnancy; duration; age of mother at birth; APGAR-score; Birth weight; breastfeeding in baby time.

**10. Head injury: **e.g. involving injuries?

### Measurement of transition

The primary outcome measure is the transition to psychosis, as defined by the CAARMS criteria [8] (see table 5).

**Table 5:** Psychosis threshold due to CAARMS

	Intensity	Frequency
Unusual Thought Content	6	4-6

Non-bizarre Ideas	6	4-6

Perceptual Abnormalities	5-6	4-6

Disorganised Speech	6	4-6

After transition to psychosis, participants are assessed with the PANSS [40] and the PSYRATS [41], to rate the severity of symptoms. They are also assessed with the SCAN 2.1 [42] interview for diagnosis according to DSM-IV criteria.

### Fidelity checks

The inter-rater reliability of the CAARMS assessments will be fine-tuned every three months in a Group wise assessment and discussion of role-played cases. The reliability of each rater is assessed by monthly independent ratings of written reports of a CAARMS interview.

The inter-rater reliability of the assessment of the fidelity of therapists in delivering the manualised therapy is fine-tuned monthly in a Group wise assessment of audio taped sessions. The fidelity of each individual therapist is assessed by a sample of five audio-taped therapy sessions rated independently by five different assessors.

### Analyses

To determine baseline balance, several variables that are proven to be risk factors will be assessed, such as severity of ARMS-symptoms [43], heredity [44], growing up in a big city, stress and unhealthy behaviour of mother during pregnancy [45], migration and feelings of discrimination [35,36] and alcohol and drug use, including cannabis use [46]. If the baseline balance is disturbed, the analyses will be corrected for risk factors for transition to psychosis.

Missing data will be imputed by EM-algorithm. EM-algorithm recovers the complete factor loadings considerably better than simple imputation techniques [47,48].

Group differences will be analysed by unadjusted chi-square. Pearson's chi-square tests will be performed to analyse gender differences between the transition group and the non-transition group, Comparisons will be analysed on intention-to-treat, using the Statistical Package for the Social Sciences (SPSS for windows, version 17). Multiple logistic regression analyses will be used to explore which factors predict transition to psychosis within the UHR-group. To examine the effects of CBT on the positive symptoms reported on the first four scales of the CAARMS, analysis of co-variance will be used.

Survival analyses will be used to measure time to transition and risk factors for developing a first episode psychosis. We will conduct Kaplan-Meier curves to explore the cumulative probability of developing psychosis with inclusion in the study as entry point and last follow-up assessment after 18 months as the end point.

We will use the Mann-Whitney test to examine if people who made a transition scored significantly higher on the CAARMS and the SOFAS compared to patients who did not make a transition. We considered p-values less than 0.05 to be statistically significant.

## Discussion

Nowadays, research increasingly focuses on early detection of patients with an At Risk Mental State. The objective is to delay the onset of psychosis or possibly even prevent a psychotic episode. A better understanding of the pre-psychotic phase is necessary to optimise preventive interventions. This study is a pilot for the implementation of early detection and intervention teams in the Netherlands and is of interest for the general mental health field, because indicated prevention for this target population is not available. The study design has a number of important strengths and limitations, as described below.

The evidence for CBT in the prevention of psychosis is still inconclusive. Only one previous study by Morrison and French [14] assessed the use of CBT in people with an ARMS. All the other studies, compared the control group with either a medication group or a combined medication and CBT group [3,12,14,15]. The present study has much more power than the above-cited study to examine the efficacy of CBT in the prevention of psychosis. Our aim is to test an intervention that aims to delay or to prevent the transition into psychosis and to reduce the persistence of ARMS. The study compares treatment as usual (TAU) with TAU plus a manualised CBT. It is partly a twin study of another large trial in the United Kingdom. A number of outcome measures are identical in both cases and we can pool the data afterwards to increase power even more. An important strength in this study is its power to generate conclusive answers on the possibility of prevention in psychosis.

There is a growing concern regarding the use of antipsychotic medication when patients potentially may not need them. Current international guidelines do not support antipsychotic medication in the prevention of psychosis. Medication can have serious side-effects and it is not yet known how long medication should be prescribed in preventing psychosis [49]. Treating people at risk with exclusively psychological means (CBT + education) could be a valuable and more benign alternative to pharmacotherapy. By providing an explanation for their odd experiences people can come to terms with these experiences. If CBT is effective in ARMS, then important goals in mental health care can be accomplished. The use of a benign time-limited intervention in the early stages will be well tolerated, while antipsychotic medication is not very well tolerated because of side-effects.

A prospective study found that 17.5% of the Dutch population has at least one psychotic feature on the CIDI [50,51]. But not all are at high risk of developing psychosis. It is not so much the experiencing of the sub clinical symptoms per se, but the distress associated with these experiences that increase the risk of transformation into psychosis. An at risk mental state becomes psychosis when the interpretation of the sub clinical symptoms becomes fixed and emotionally stressful. Normalising these experiences and giving realistic explanations might reduce the emotional arousal as a result of these disturbing experiences and may prevent people from adhering to a catastrophic delusional understanding of their unusual experiences. The exposure to other risk factors, such as urbanisation, trauma or cannabis use also increases the probability of psychotic transition [50,51]. The study can help to build a neuropsychiatric model of ARMS and inform whether cognitive biases are indeed causally involved in the formation of delusions.

All the intervention studies compared a specific intervention group with monitoring assignments. This study, on the other hand, compares two active treatment conditions; it can determine the specificity of the CBT intervention on cognitive biases. The TAU condition targets the reduction of symptoms and emotional discomfort of the 'co morbid' disorders and the CBT condition additionally targets cognitions about the sub clinical symptoms and the cognitive biases that are involved in delusion formation. If psychosis formation is driven by emotional arousal, than both treatment conditions will reduce the transition rate. In that case a specific intervention is obsolete, as treating the co morbid disorder will take away enough emotional arousal to prevent a transition into psychosis. A weakness is that we cannot demonstrate the efficacy of treating the co morbid disorder on preventing transitions, because a no-treatment control group is lacking.

A possible strength is the two-stage screening procedure used to detect subjects at risk. Screening the entire help-seeking population referred to community mental health services with a two-stage strategy, can potentially detect more ARMS patients by uncovering at risk cases that would normally never be referred. Many pre-psychotic symptoms are not recognised during the regular referral process. Because we do not want to miss any patients, we probably will not only detect late prodromal patients, but also early prodromal patients. Therefore, this two-stage screening method will detect more ARMS patients than the existing referrals to the specialized clinic, but probably at the expense of more false positives. The referred patients are more likely to be late prodromal than the people recruited by screening. Screening with a highly specific screening tool eliminates most of those not at risk in the first stage. The selected percentage undergoes the second stage of screening with the CAARMS interview. In this enriched sample of (more) psychosis-prone individuals the assessment instruments will probably have a better predictive value. In concordance with previous research [50] we expect about 3.5% of the help-seeking population will have psychotic-like experiences and will have an at-risk mental state. In the selected group we expect the transition rate to be 25 to 35 percent over an 18 month period. If this two-stage screening process proves to be successful, this type of screening could be implemented in routine mental health care.

Another strength of our study is that it has a strong external and internal validity. Since this trial is implemented in a mental health setting, the results will immediately be relevant for clinical practice. All participants receive treatment in a regular outpatient treatment centre in the same way they would have received treatment if they were not enrolled in the study. The internal validity is fine-tuned by supervision of the therapists every two weeks. The researchers and research assistants have supervision every three months. The internal validity is measured by the inter-rater reliability of the CAARMS; the therapy is monitored regularly and reliable and valid instruments are used.

In conclusion, the study may be of great importance for the development and implementation of early detection and prevention of psychosis. We expect that the two-stage screening method, as compared to traditional referral to a specialist clinical psychosis centre, will contribute to the detection of more UHR patients. Additionally, educating people at risk on cognitive biases and treating these with CBT could be a successful strategy. Most people with ARMS are involved in a search for a plausible explanation for their unsettling experiences. Normalising these experiences, providing benevolent explanations and limiting exposure to known risk factors will probably reduce the arousal and prevent people from adhering to a catastrophic delusional explanation for their strange experiences. We have reason to hope that the intervention will delay or prevent the onset of psychosis and lower the burden and anxiety caused by sub clinical symptoms.

## Competing interests

The authors declare that they have no competing interests.

## Authors' contributions

All authors contributed to the design or the implementation of the study. MvdG, PC and DL designed the study. MvdG is the principal investigator of the study. JR and SD drafted the manuscript, were responsible for the logistics within the departments of the psychiatric centres and took care of the recruitment of participants and assessment and diagnoses. RK and HI looked after the recruitment of participants and data collection. DN is one of the therapists in the study, participated in the coordination and supervision of the PhD-students and helped to draft the manuscript. LW and RK made the recruitment possible in his/her psychiatric centre and helped to draft the manuscript. PC, LW, PhD, DL and MvdG will act as Quality Assurance Committee for this trial. MvdG prepared the educational manual on cognitive biases and odd experiences. All authors provided comments, read and approved the final manuscripts.

## Authors' informations

Judith Rietdijk (MSc) is a psychologist and PhD-student at the VU University and EMGO Institute of Health and Care Research in Amsterdam, the Netherlands. Sara Dragt (MD) is a PhD-student at the Academic Medical Centre Amsterdam (AMC), the Netherlands. Rianne Klaassen (MD) is a consultant child and adolescent psychiatrist in Leiden, working for GGZ Rivierduinen, and a PhD-student at the AMC. Helga Ising (MSc) is a psychologist and PhD-student at the Parnassia Psychiatric Institute in The Hague and EMGO Institute of Health and Care Research in Amsterdam, the Netherlands. Dorien Nieman (PhD) is a clinical psychologist and post doc researcher at the Department of Psychiatry, AMC University of Amsterdam, the Netherlands. Lex Wunderink (PhD) is psychiatrist at GGz Friesland, Leeuwarden, the Netherlands. Philippe Delespaul (PhD) is head of the Psychosis Department of the Mondriaan Psychiatric Institute and associate professor at the Department of Psychiatry and Neuropsychology, Maastricht University, the Netherlands. Pim Cuijpers (PhD) is professor of Clinical Psychology and head of the Department of Clinical Psychology at the VU University in Amsterdam. He is also Vice-Director of the EMGO Institute of Health and Care Research in Amsterdam, the Netherlands. Don Linszen MD (PhD) is professor of Psychiatry, AMC University of Amsterdam, head of the Adolescent psychosis clinic, Department of Psychiatry, AMC University of Amsterdam, the Netherlands. Mark van der Gaag (PhD) is head of Psychosis Research at the Parnassia Psychiatric Institute in The Hague and professor of Clinical Psychology at the VU University and the EMGO Institute in Amsterdam, the Netherlands.
